# Human cortical activity evoked by contextual processing in attentional orienting

**DOI:** 10.1038/s41598-017-03104-1

**Published:** 2017-06-07

**Authors:** Shuo Zhao, Chunlin Li, Shota Uono, Sayaka Yoshimura, Motomi Toichi

**Affiliations:** 1 Faculty of Human Health Sciences, Graduate School of Medicine, Kyoto University, Kyoto, 606-8507 Japan; 2 International Research Fellow of the Japan Society for the Promotion of Science, Tokyo, 102-0083 Japan; 3 Organization for Promoting Neurodevelopmental Disorder Research, Kyoto, 606-8392 Japan; 4 School of Biomedical Engineering, Capital Medical University, Beijing, 100069 China; 5 Department of Neurodevelopmental Psychiatry, Habilitation and Rehabilitation, Graduate School of Medicine, Kyoto University, Kyoto, 606-8507 Japan

## Abstract

The ability to assess another person’s direction of attention is paramount in social communication, many studies have reported a similar pattern between gaze and arrow cues in attention orienting. Neuroimaging research has also demonstrated no qualitative differences in attention to gaze and arrow cues. However, these studies were implemented under simple experiment conditions. Researchers have highlighted the importance of contextual processing (i.e., the semantic congruence between cue and target) in attentional orienting, showing that attentional orienting by social gaze or arrow cues could be modulated through contextual processing. Here, we examine the neural activity of attentional orienting by gaze and arrow cues in response to contextual processing using functional magnetic resonance imaging. The results demonstrated that the influence of neural activity through contextual processing to attentional orienting occurred under invalid conditions (when the cue and target were incongruent versus congruent) in the ventral frontoparietal network, although we did not identify any differences in the neural substrates of attentional orienting in contextual processing between gaze and arrow cues. These results support behavioural data of attentional orienting modulated by contextual processing based on the neurocognitive architecture.

## Introduction

The ability to assess another person’s direction of attention is paramount in social communication. For example, we are able to identify a person’s focus based on their eye gaze, thus enabling an understanding of other people’s inner state (such as thoughts, beliefs, and desires)^1^. Similar to eye gaze, non-social stimuli also play important roles in influencing attention, such as an arrow on a road sign. However, compared with eye gazes, non-social directional stimuli are not helpful when making conclusions regarding someone’s cognitive state, such as speculating about what a person wishes to do.

Over the past two decades, cognitive psychologists have focused on comparing the role of directional gaze and arrow cues in attentional orienting. These studies have typically investigated attentional orienting based on gaze and arrow cues using a modified version of Posner’s cueing paradigm^2^. For example, Friesen and Kingstone^3^ presented non-predictive gaze cues at the centre of a screen prior to the presentation of a peripheral target (right or left). Before the onset of the target, a centrally presented directional cue (e.g., eye gaze) appears on screen. Under valid conditions, the cue will accurately indicate the subsequent target location, whereas under invalid conditions, the cue will indicate the opposite location. A rapid response to a validly cued target indicates an allocation of attention (i.e., orienting) to the target location prior to target onset. In contrast, a delayed response to an invalidly cued target occurs when the onset of the target at the opposite location, indicating a reorienting of attention to the target. Previous studies^4–9^ have commonly demonstrated that arrow cues automatically trigger attentional shifts in the same manner as gaze cues. These studies have demonstrated that both gaze and arrow cues trigger attentional shifts when they are counterpredictive of a target location^7^, facilitate response time when discriminating the target following the cue^6^, have comparable sensitivity to object-based selection^4, 5, 8^ and the stimulus onset asynchrony between the cue and target^9^.

Recent neuroimaging studies regarding attentional orienting have attempted to investigate differences in cortical activity between gaze and arrow cues. These studies have focused on two attentional networks (reviewed by^10, 11^). The dorsal frontoparietal network, with regions centred around the intraparietal sulcus (IPS), superior parietal lobule (SPL)/Brodmann’s area (BA)5, 7, and frontal eye field (FEF)/BA8, may be responsible for orienting of attention to a validly cued target in the cueing paradigm, but also for reorienting attention to an invalidly cued target^10, 11^. The ventral frontoparietal network, with regions centred on the temporoparietal junction (TPJ)/BA39, 40, 22 and ventral frontal cortex (VFC)/BA44, 45, 47 (including parts of the middle frontal gyrus (MFG) and inferior frontal gyrus (IFG)), may only be responsible for reorienting attention. Most previous studies^12–18^ have demonstrated that the differences in cortical activity associated with social gaze and arrow cues are quantitative rather than qualitative, although some studies^19–21^ have reported evidence suggesting different mechanisms for these cues. For example, Tipper *et al*.^17^ reported attentional orienting to both eye gaze and arrow cues engaged extensive dorsal and ventral frontoparietal networks, but the magnitude of activation differed between these networks. However, these studies only examined the differences between gaze and arrow cues under simple conditions (e.g. a dot or letter as the target). Given that Birmingham and Kingstone^22^ suggested that the apparent difference in attentional orienting between gaze and arrow cues might be distinguished only when the cues were embedded in a rich environment, it is thus important to examine the differences between gaze and arrow cues under more complex conditions.

Some studies have highlighted the importance of contextual processing (i.e., the semantic congruence between the cue and target) in attentional orienting when using arrows or eye gaze as cues. Previous studies^23, 24^ have demonstrated that attentional orienting is facilitated through contextual processing when using arrows as cues. For example, Ristic *et al*.^23^ examined attentional orienting based on whether facial gaze and arrow cues could be triggered through the contextual processing of cue-target colour contingencies. The results indicated that attentional orienting elicited by an arrow rather than an eye gaze was sensitive to colour-congruent target stimuli; an attentional orienting effect for blue arrows was only evident for blue targets. However, other studies^25–27^ have demonstrated that attentional orienting with facial gaze was facilitated through a strongly contextual relationship between the cue and target when there was congruence in meaning between the cue and target. For example, Bayliss *et al*.^25^ reported that compared with disgusted faces, the gaze direction of happy faces more effectively oriented attention to pleasant targets. These findings indicated that participants could employ contextual information in attentional orienting by arrows or eye gaze cues to effectively capture important information, although the context effect might be observed only when targets are presented at a specific context of colour and emotion for gazes and arrows. These findings raised a question regarding whether attentional orienting differs between eye gaze and arrow cues when these cues were influenced through contextual processing.

At a neural level, researchers have shown activity in the TPJ and superior temporal sulcus (STS)/BA21, 22 associated with contextual processing in attention. Geng and Vossel^28^ reviewed previous evidence, indicating that the TPJ (anatomically, the TPJ is strictly defined as the cortex at the intersection of the posterior superior temporal, supramarginal, and angular gyri) was engaged in terms of “contextual updating” in attention. For example, Weidner *et al*.^29^ demonstrated that cortical activity in TPJ increased when the contextual processing of the relationship between the cue and target was incongruent as opposed to congruent (i.e., when the target-defining dimension (orientation or colour) was incongruently rather than congruently cued). Moreover, Noppeney *et al*.^30^ observed that the activity in STS increased when a sound or speech target was incongruent (e.g., a car picture paired with the spoken word ‘owl’) as opposed to congruent (e.g., a cat picture paired with the spoken word ‘cat’) with prior visual information. This finding indicated that context also modulated the activity of STS. Consistently, when a strong relationship was established between the target word and a word sound that had been previously presented, the results showed the enhanced activation for thematically related categories (e.g., picture + frame) and response suppression for taxonomically related categories (e.g., chair + armchair) in the left STS^31^. In the present study, we focused on these brain regions to examine the influence of neural systems in relation to gaze and arrow cues through contextual processing focused on the relationship between the cue and target in attentional orienting.

In the present study, we examined the neural activity of attentional orienting with social gaze and arrows as cues using Posner’s cueing paradigm. Based on a previous study^27^, two sounds (a social voice and a tone) were manipulated as targets to determine the contextual relationship between cue and target; that is, social gaze and social voice and arrow and tone as congruent meaning conditions, and social gaze and tone and arrow and social voice as incongruent meaning conditions. The aims of this study are as follows: (1) We first wanted to examine whether the influence of neural activity in TPJ and STS differed between gaze and arrow cues in response to contextual processing of the relationship between cue and target. (2) Furthermore, given that previous studies^10, 11^ have characterised the functional mechanisms of the orienting and reorienting of attention (i.e., valid and invalid conditions) in dorsal and ventral frontoparietal networks, respectively, we considered it important to specifically investigate these functional mechanisms and how they were modulated through the contextual processing of the relationship between cue and target. Specifically, if different neural activity was observed for gaze and arrow in response to the contextual processing of cue-target, we would subsequently examine whether the neural activity for attentional orienting differed with contextual processing between gaze and arrow cues at the dorsal and ventral frontoparietal networks, respectively. In contrast, if no difference between gaze and arrow was evident, we would then examine only the influence of neural activity for attentional orienting by contextual processing in both gaze and arrow cues in these two attentional networks.

## Methods

### Participants

This research was approved by the local ethics committee of Capital Medical University, Beijing, China. No foreseeable risk to the participants was present, and personal identifying information was not collected. Participants provided written informed consent and background information. All procedures complied with the ethical standards of the 1964 Declaration of Helsinki regarding the treatment of human participants in research. In total, 22 volunteers (9 women, 13 men; mean ± SD age, 22.95 ± 2.61 years) participated. All participants were right-handed, as assessed by the Edinburgh Handedness Inventory^32^, and had normal or corrected-to-normal visual and auditory acuity.

### Stimuli

**Figure 1:**
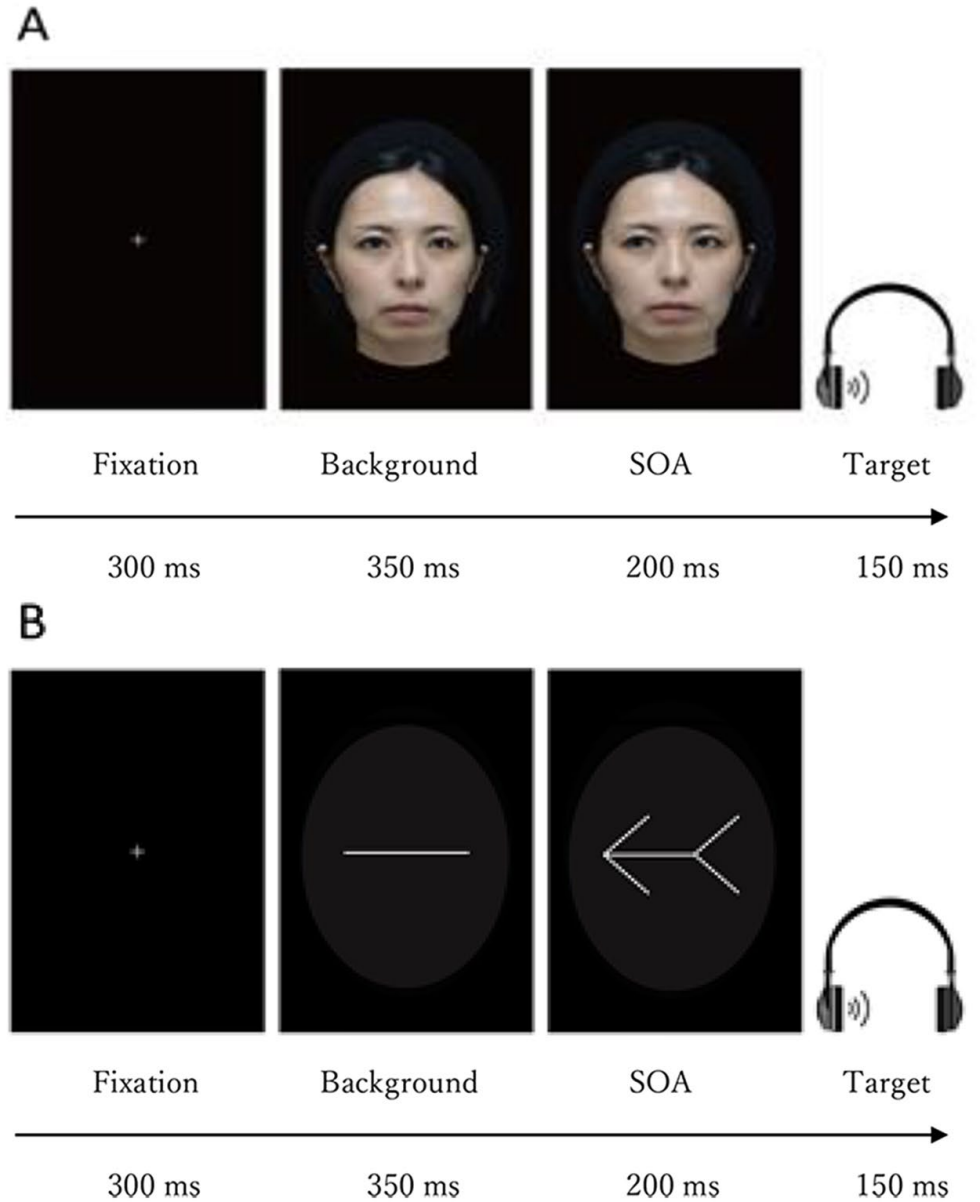
Illustration of the stimulus presentation.

Visual and auditory stimuli were almost identical to those used in a previous behavioural study (at a sound level comfortable to each participant)^27^. Previous studies have demonstrated that female faces are less resemblance to angry expressions than male faces, and male faces are perceived as less likeable^33, 34^ and more powerful^35^. To avoid any differential influence of expression (e.g., anger), a Japanese female face with neutral expressions was used for this task (Fig. 1A). The image was obtained from a previous study^36^, in which the emotional intensity of facial stimuli with neutral expressions was assessed. The results confirmed that these facial images were considered neutral rather than emotional. Based on these findings, it is reasonable to propose that the female face image in the present study conveyed neutral facial expressions. Moreover, three versions of each face were produced: one version with a direction of gaze straight ahead, another version with the pupils averted leftward, and a third version with the pupils averted rightward. The faces measured approximately 4.7° wide and 6.9° high. For the arrow cue, a symmetrical arrow was presented as the cue stimulus, with an arrowhead at one end and a tail at the opposite end. The arrows measured 4.7° in width by 1.7° in height and were light grey.

Furthermore, two types of auditory stimuli were presented as targets. One type was sampled from a woman: an/i/voice sound (F0 frequency of 300 Hz), which is similar to the /iy/sound in English. The other type was a pure tone of similar frequency to the F0 voice (300 Hz), which was produced using the Audacity software package (ver. 1.3.13; Audacity store.com). The duration of the stimulus presentation was 150 ms.

### Apparatus

These stimuli were generated on a computer and presented to the participants via a custom-built, magnet-compatible audio-visual system during magnetic resonance (MR) scanning. To attenuate the acoustic noise that accompanies fMRI (functional magnetic resonance imaging) scanning, shooting earmuffs were used. Participants viewed visual stimuli on a back-projection screen. The auditory stimuli were identical to those presented in a previous study^37^ via an air-conductive tube to participants. Presentation software (ver. 10.2; Neurobehavioral Systems) was used to generate auditory and visual stimuli on a Windows computer. In addition, the participants generated their responses using a keypad (Current Designs Inc., Philadelphia, PA, USA).

### Procedures

The sequence of stimulus presentation is shown in Fig. 1. For each trial, a fixation cross was initially presented for 300 ms in the centre of the screen. A neutral stimulus with a straight eye gaze (gaze trail) or transverse lines (arrow trail) was subsequently presented at the location. After 350 ms, a cue stimulus (gaze or arrow) in the right or left direction was presented in the centre of the screen. The stimulus onset asynchrony (SOA) between the auditory target and cue was fixed to 200 ms. Subsequently, an auditory stimulus target (voice or tone sound) was presented in the left or right ear for 150 ms through headphones. Consistent with previous studies^37–39^, the participants were asked to answer quickly and precisely whether or not they heard the auditory target on the left or right side of the headphones by pressing the corresponding key on the switch keypad using their dominant index or middle fingers, respectively. Response times (RT) were measured in each trial. A standard procedure for the Posner’s cueing paradigm removed cue stimuli before a target stimulus appeared on the display. However, when using a facial gaze as a cue, many studies e.g. refs 40 and 41 also implemented a modified cueing paradigm in which the cue remained on the screen until a response was obtained or a set time had elapsed. For this study, we designed a contextual processing condition between the cue and target. To establish a strong and obvious contextual relationship between the cue and target, the cue remained until a response was obtained or 1000 ms had elapsed. The targets appeared randomly on the same or opposite side of the cue direction when the cue was directed left or right. The target appeared at the cued location in 50% of the trials. The participants were told that the cue did not predict the target location and were instructed to fixate on the centre of the screen in each trial.

The fMRI analysis relied on a within-subject three factorial design, with the cue condition (gaze or arrow), context condition (i.e., the congruence of meaning between the cue and target, which could be congruent (social gaze and social voice or arrow and tone) or incongruent (social gaze and tone or arrow and social voice)), with validity condition (valid or invalid) as the repeated factors. Sixty trials were performed under each condition. Our experimental design was based on a mixed block/event-related paradigm, facilitating a more complete utilisation of the BOLD signal and enabling a deeper interpretation of how the regions of the brain function on multiple timescales^42^. Consistent with a previous study^43^, alternating blocks of experimental trials of cue condition and blocks of baseline were presented. Within the condition blocks, congruence trials were presented in a pseudorandom event-related distribution.

### MRI acquisition

The images were acquired using a 3.0-T Trio Tim Scanner-vision whole-body MRI system (Siemens, Erlangen, Germany) to measure activation using a head coil. The functional images comprised 33 consecutive slices parallel to the anterior-posterior commissure plane, covering the entire brain. A T2*-weighted gradient-echo planar imaging (EPI) sequence was used with the following parameters: TR = 2000 ms, TE = 30 ms, flip angle = 90°, field of view = 220 × 220 mm, matrix size = 64 × 64, and voxel size = 3.4 × 3.4 × 3.5 mm^3^. The slices covered most of the brain, including the entire temporal cortex, but excluding the most inferior parts of the cerebellum. We also acquired high-resolution isotropic T1-weighted images (TR = 1900 ms, TE = 2.52 ms, flip angle = 9°, field of view = 250 × 250 mm, 176 sagittal slices, voxel size = 1 × 1 × 1 mm^3^).

### Behavioural data analysis

The data were analysed using the SPSS software package (ver. 21.0). Incorrect responses (1.76% of the trials) and RT of less than 100 ms or more than 1000 ms were excluded from the RT analysis (1.18% of the trials), and trials in which a response occurred prior to the target onset were also excluded. The mean RT under conditions was calculated for each participant. The mean RT was analysed using a three-way analysis of variance (ANOVA) with cue (gaze, arrow), context (congruent, incongruent), and validity (valid, invalid) as within-participant factors. To examine whether an interaction was significant, if present, a follow-up simple main effect (i.e. assessing the effect of each independent variable at each level of the other independent variable) analysis was conducted to interpret the result.

### Image data analysis

Data preprocessing and statistical analyses were performed using the Statistical Parametric Mapping software package (SPM12; Wellcome Department of Cognitive Neurology, London, UK; http://www.fil.ion.ucl.ac.uk/spm/software/spm12) implemented in MATLAB 2013b (Math Works). The functional images from each run were realigned using the first scan as a reference to correct for head movements. The movement parameters generated during spatial realignment indicated that all subjects moved less than 2 mm during the course of the trial. The T1 anatomical image was preprocessed using an intensity inhomogeneity correction. Then, T1 anatomical images were coregistered to the first scan of the functional images. Next, the coregistered T1 anatomical image was normalised to the Montreal Neurological Institute space using a unified segmentation-spatial normalisation approach^44^. The parameters from this normalisation process were subsequently applied to each of the functional images. Finally, these spatially normalised functional images were resampled to a voxel size of 2 × 2 × 2 and were spatially smoothed in three dimensions using an 8-mm full-width-at-half-maximum Gaussian kernel.

We used random-effects analyses^45^ to identify significantly activated voxels exhibiting interesting effects. First, we performed a single-subject analysis^46^. The BOLD response was modelled as the neural activity, convolved with a canonical haemodynamic response function (HRF), which yielded regressors in a general linear model (GLM) for each condition. We used a high-pass filter comprising a discrete cosine basis function with a cut-off period of 128 to eliminate the artefactual low-frequency trend. To correct the global fluctuation related to motion artefacts, global scaling was conducted. Serial autocorrelation, assuming an AR (1) (first-order autoregressive) model, was estimated from the pooled active voxels with a restricted maximum likelihood procedure and used to whiten the data and design matrix^47^.

The contrast images from the first-level analyses from all subjects were subsequently used for the second-level group statistics. First, for each participant, the data were best fitted at every voxel using a combination of effects of interest. These data were delta functions representing the onsets of the eight conditions, given by the crossing of our 2 × 2 × 2 factorial design: cue (gaze, arrow) × context (congruent, incongruent) × validity (valid, invalid), convolved with the SPM12 haemodynamic response function. Second, based on the behavioural results, a 2 × 2 × 2 (cue × context × validity) factorial ANOVA was used to investigate the relationship between behavioural results and brain activation. Based on a methods analysis^48^, the statistical maps exhibited a spatial extent threshold at p < 0.05, family-wise error (FWE)-corrected for multiple comparisons, and an intensity threshold at p < 0.001, uncorrected for multiple comparisons at the whole-brain level was used to protect against false-positive activations. The peak voxels of clusters exhibiting reliable effects are reported in MNI coordinates. We had an a priori hypothesis regarding the activity of contextual processing in TPJ and STS, and the influence of contextual processing in dorsal and ventral frontoparietal networks. Based on anatomical masks using the WFU Pickatlas tool, a small-volume correction was also employed to the a priori regions of interest, attributed to the anatomical structures in left/right hemisphere of the STS with BA21, 22, the IPS and SPL with BA5, 7, the FEF with BA8, and the IFG with BA44, 45, 47, separately. Consistent with the whole-brain level, we used small-volume correction at a voxel spatial extent threshold at p < 0.05, FWE corrected, and an intensity threshold at p < 0.001, uncorrected for multiple comparisons. Finally, to quantify neural responses with the influence of attentional orienting under context conditions, we used the MarsBaR software package^49^ to extract percentage changes in BOLD signals for congruent and incongruent contexts under valid and invalid conditions, averaged across voxels with given regions of interest (ROI) using spheres with a radius of 8 mm. Then, the means of the percent signal change (PSC) between the conditions were compared using repeated-measures ANOVA. All statistics were calculated using the SPSS software package (ver. 21).

## Results

### Behavioural results

The data pertaining to errors did not reveal any significant main effect or interaction (all *p* > 0.05), thus indicating that the participants suffered no speed-accuracy trade-off (Table 1).

**Table 1:** Mean response times (ms), standard deviations, and percent errors (%E) as a function of cue, context, and validity.

Cue	Validity	Context					
		Congruent			Incongruent		
		*M*	*SD*	%E	*M*	*SD*	%E
Gaze	Valid	328.3	28.3	0.15	330.3	27.9	0.15
	Invalid	344.0	26.7	0.31	356.2	26.7	0.34
Arrow	Valid	337.1	26.3	0.15	343.4	27.2	0.12
	Invalid	355.9	25.3	0.27	368.8	26.7	0.26

**Figure 2:**
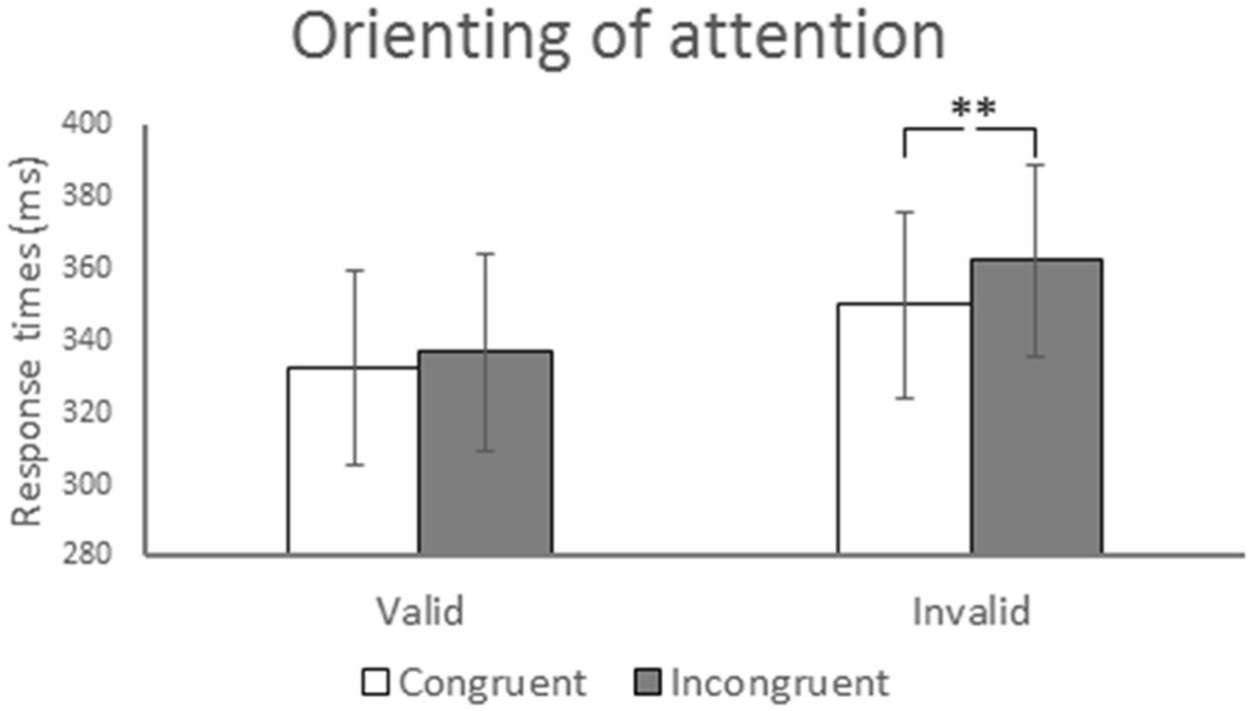
Response times (RT) results in attentional orienting. Mean (with SE) RT presented for valid and invalid conditions as a function of a cue type condition (gaze or arrow). **p < 0.01.

The mean RT under each condition are listed in Table 1, and the mean differences in RT between the invalid and valid conditions are shown in Fig. 2. A three-factor repeated-measures ANOVA was used to analyse the RT. The analysis revealed a main effect of cue (*F* (1, 21) = 12.412, *p* = 0.002, *η*_*p*_^*2*^ = 0.371), with faster responses under the eye gaze (339.7 ms) versus arrow (351.3 ms) condition. In addition, we also observed a significant main effect of context (*F* (1, 21) = 8.213, *p* = 0.009, *η*_*p*_^*2*^ = 0.281), with faster responses under congruent (341.3 ms) versus incongruent (349.7 ms) conditions, and validity (*F* (1, 21) = 25.247, *p* < 0.001, *η*_*p*_^*2*^ = 0.546), with faster responses under valid (334.8 ms) versus invalid (356.2 ms) conditions.

A significant interaction of context × validity was observed (*F* (1, 21) = 4.907, *p* = 0.038, *η*_*p*_^*2*^ = 0.189), but no significant interaction was detected for cue × context (*F* (1, 21) = 0.166, *p* = 0.688, *η*_*p*_^*2*^ = 0.008), cue × validity (*F* (1, 21) = 0.048, *p* = 0.828, *η*_*p*_^*2*^ = 0.002), or cue × context × validity (*F* (1, 21) = 0.108, *p* = 0.746, *η*_*p*_^*2*^ = 0.005).

The *post hoc* test revealed a significant difference between context conditions under invalid conditions (*p* = 0.004) but not under valid conditions (*p* = 0.182) with a faster response for congruent (349.9 ms) versus incongruent (362.5 ms) under invalid conditions, indicating an RT benefit for targets that match the context (e.g. social) of the cue under invalid conditions but not under valid conditions. This result suggests that the disengagement of attention from cued locations is facilitated through contextual processing. These findings demonstrated that attentional orienting is modulated through contextual processing only under invalid conditions, enabling the investigation of the neural substrates underlying the behavioural response of the contextual processing between the cue and target in attentional orienting induced by gaze and arrow cues.

### Supplementary analysis of the influenced by the gender

Because previous studies have reported that gaze-triggered orienting is different between genders (e.g., refs 50 and 51), we added gender (male, female) as between-participant factor to supplement the influence of the gender based on the main (3-way) ANOVA of RT data analysis, although 22 participants were recruited with gender unbalance including 9 women and 13 men. The results found a significant interaction between gender and the validity (*F* (1, 20) = 9.591, *p* = 0.006, *η*_*p*_^*2*^ = 0.324) but not between gender and other factors (all *F* (1, 20) ≤ 2.54, *p* > 0.1). However, the *post hoc* test did not reveal a difference between genders under valid or invalid conditions (both *p* > 0.1), although a faster response was observed for valid compared with invalid conditions in both male (323.5 vs. 335.6 ms, *p* = 0.017) and female (351.1 vs. 386.0 ms, *p* < 0.001) participants. Thus, the results of the present study suggest that attentional orienting through contextual processing was not influenced by the gender of the participants.

### fMRI results

Next, based on the behavioural results, we investigated the patterns of brain activation associated with cross-modal attention. In the primary analysis, we performed 2 cue conditions (gaze, arrow) × 2 context conditions (congruent, incongruent) × 2 validity conditions (valid, invalid) repeated-measures ANOVA.

### Main effects of cue, validity, and context

In a whole-brain analysis, the gaze trials evoked significantly greater activity than arrow trials in a many clusters of voxels. One of these clusters included the fusiform gyrus (BA 19), extending from the extra-striate visual areas into the occipital and temporal cortices (Supplementary Fig. S1, Table S1). In contrast, greater activity for arrow than for gaze trials was observed in the right hemisphere of temporal lobe, including the middle temporal gyrus (BA 37), and the left hemisphere of occipital lobe, including the middle occipital gyrus (BA 19) (Supplementary Fig. S2, Table S2). These results were consistent with previous evidence^21^, thus indicating that gaze versus arrow cues increased activation in various occipital and temporal areas, whereas the reverse contrast evoked activation in occipital regions.

Furthermore, in a whole-brain analysis, invalid gaze and arrow cues evoked a significantly larger response than valid gaze and arrow cues in the left frontal hemisphere and the limbic system, including the inferior and middle frontal gyrus, and the anterior cingulate (Supplementary Fig. S3, Table S3). These results were also consistent with those of a previous study^16^ that revealed common activity in the IFG by conjunction analyses to response gaze and arrow cues in attentional orienting. However, activation was not observed by valid versus invalid gaze and arrow cues.

**Figure 3:**
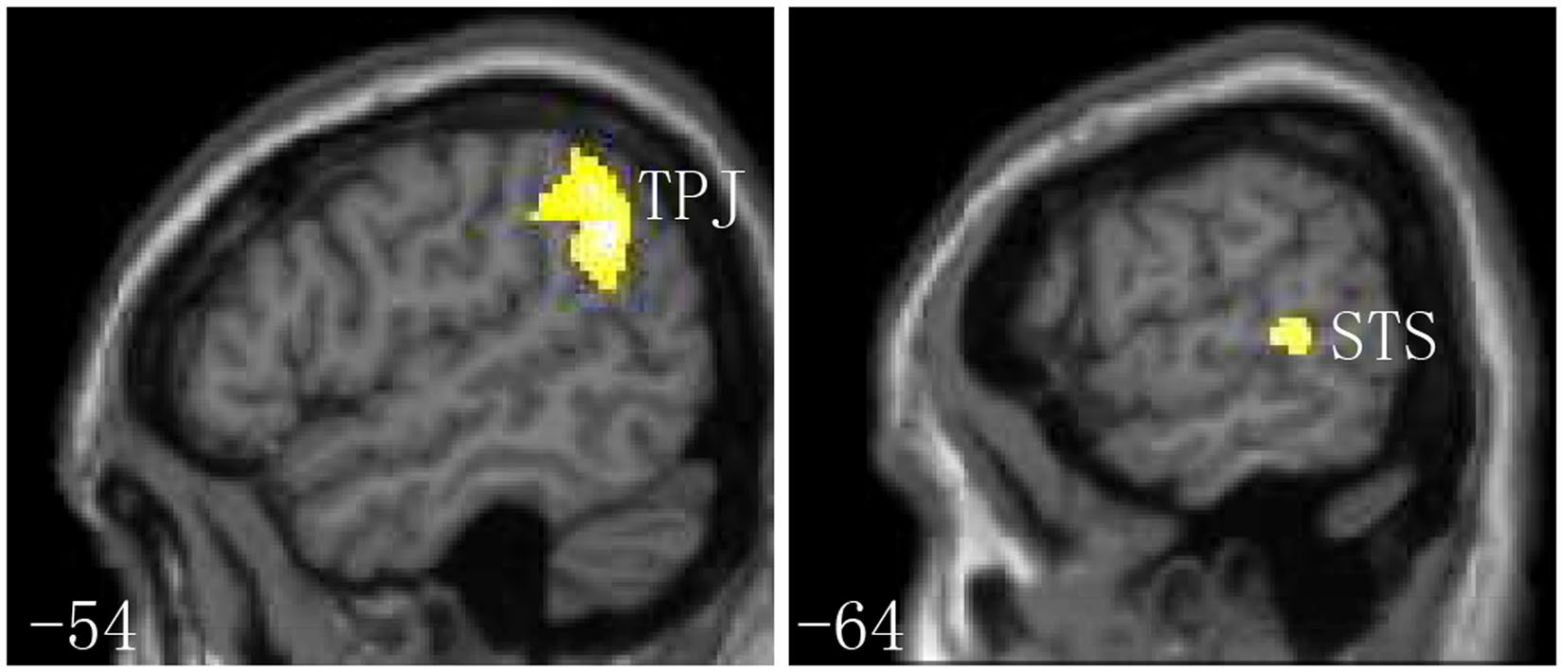
In response to incongruent versus congruent context conditions, exploratory whole-brain analysis indicating that the left TPJ is significantly activated, and small-volume-correction analysis showing that the left STS is significantly activated based on an anatomical mask. A voxel-wise spatial extent threshold *p* < 0.05, FWE-corrected, and an intensity threshold *p* < 0.001, uncorrected, were used.

To highlight the neural underpinnings of RT modulated through contextual processing, we examined the difference between congruent and incongruent contextual meanings of the cue-target. The results of the whole-brain analysis indicated that the congruent condition evoked a significantly smaller response than the incongruent condition in the left hemisphere parietal, including TPJ (BA 40). Additionally, anatomical region-based small-volume corrections revealed significant activation in the temporal lobe, including STS (BA21/22). (Fig. 3, Table 2) These findings indicated that activity in left TPJ and STS regions was associated with the contextual processing of the cue-target, but this activation was not observed in the congruent versus the incongruent condition. Furthermore, to examine the differences between gazes and arrows in contextual processing, we investigated whether the neural activity in these regions differed between gaze and arrow cues. The results revealed no significant difference between gaze and arrow cues, indicating that comparable neural activity was elicited by contextual processing between gaze and arrow, thereby influencing valid and invalid orienting within attentional orienting. Next, we assessed the cue condition, focusing on its influence in contextual processing for both gaze and arrow cues in attentional orienting.

**Table 2:** Main effect of incongruent condition: incongruent > congruent.

Side	Area	Region	BA	Coordinates			Z-value	P (FWE)	P (FWE)	P (uncorr)	Cluster size
				x	y	z		(cluster level)	(peak level)	(peak level)	(mm^3^)
Exploratory whole-brain analysis											
L	Parietal	Temporoparietal junction	40	−54	−50	34	4.696	0.001	0.021	0.000	819
Small-volume corrections analysis											
L	Temporal	Superior temporal sulcus	21/22	−64	−34	6	3.943	0.028	0.005	0.000	41

BA = Brodmann’s area; FWE = family-wise error; a voxel-wise spatial extent threshold at *p* < 0.05, FWE corrected, and an intensity threshold at *p* < 0.001, uncorrected.

### Interaction of context and validity conditions

**Figure 4:**
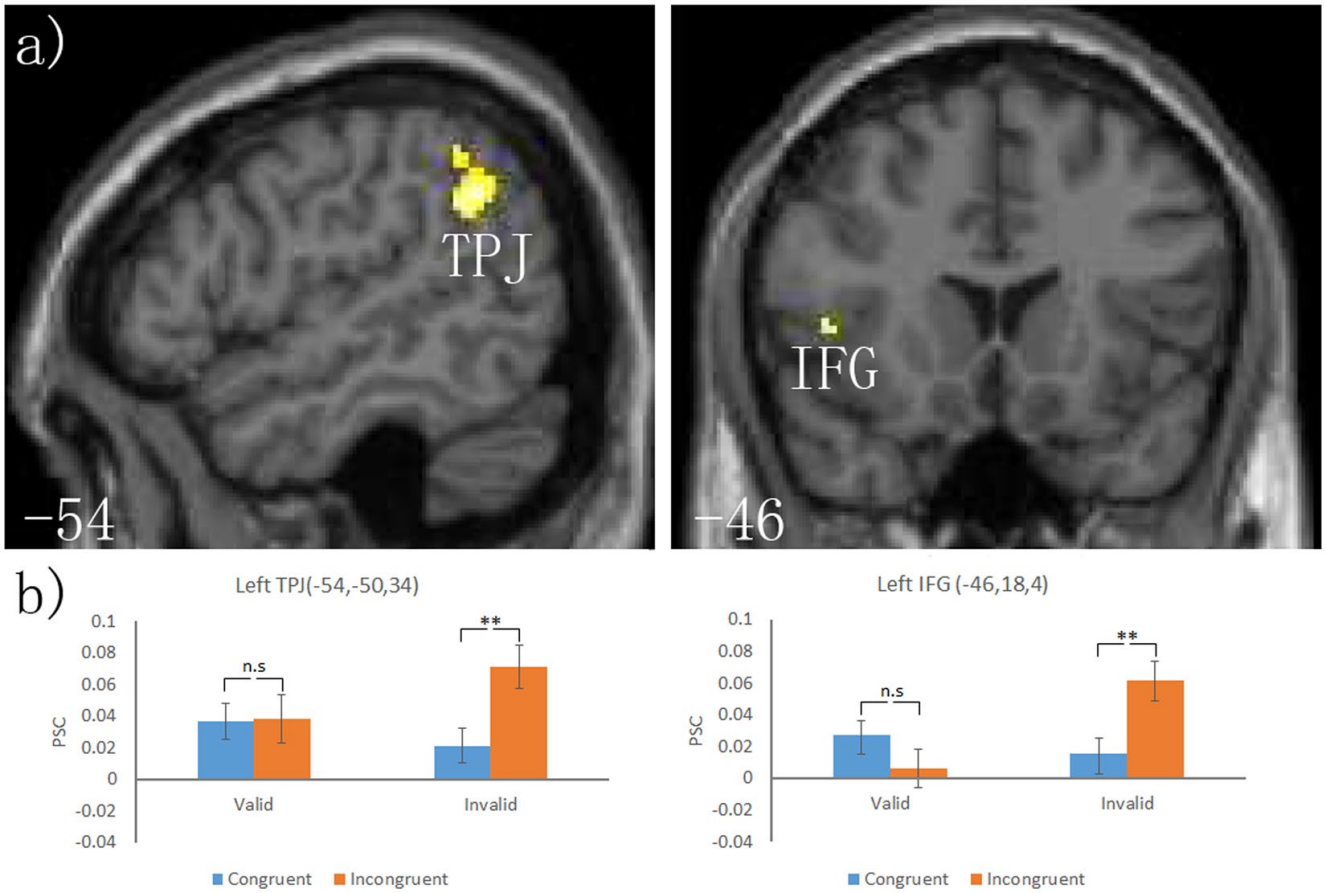
(**a**) In response to the interaction between context and validity conditions, exploratory whole-brain analysis showing left TPJ significantly activated, and small-volume-correction analysis showing left IFG significantly activated based on an anatomical mask. A voxel-wise spatial extent threshold *p* < 0.05, FWE corrected, and an intensity threshold *p* < 0.001, uncorrected, were used. (b) Mean (±SE) and percent signal changes (PSC) in the left hemisphere TPJ and IFG regions are shown. These areas are overlaid on the mean normalised structural MRI from all subjects in this study. n.s: *p* > 0.05; **p < 0.01.

A 2 (context: congruent, incongruent) × 2 (validity: valid, invalid) ANOVA was performed to investigate the influence of activation by the contextual relationship of cue-target in attentional orienting networks. The results of the whole-brain analysis revealed a significant interaction in left hemisphere TPJ (BA 40). Additionally, anatomical region-based small-volume corrections revealed significant activation in the left hemisphere IFG (BA 47) regions (Fig. 4, Table 3).

**Table 3:** Interaction between context and validity conditions.

Side	Area	Region	BA	Coordinates			Z-value	P (FWE)	P (FWE)	P (uncorr)	Cluster size
				x	y	z		(cluster level)	(peak level)	(peak level)	(mm^3^)
Exploratory whole-brain analysis											
L	Parietal	Temporoparietal junction	40	−54	−50	34	3.962	0.01	0.345	0.000	391
Small-volume corrections analysis											
L	Frontal	Inferior frontal gyrus	47	−46	18	4	3.356	0.04	0.025	0.000	5

BA = Brodmann’s area; FWE = family-wise error; a voxel-wise spatial extent threshold at *p* < 0.05, FWE corrected, and an intensity threshold at *p* < 0.001, uncorrected.

### ROI analysis

The results of the interaction were expanded using an ROI-based analysis. Figure 4 and Table 4 present the location and pattern of the response in all ROIs in which a signal change was extracted. These responses were located in left TPJ and IFG regions. The PSC in these regions was analysed using a 2 (context: congruent, incongruent) × 2 (validity: valid, invalid) repeated-measures ANOVA. A significant interaction was observed in the left TPJ and IFG regions. Moreover, the post hoc test revealed that PSC was smaller when the contextual meaning of cue-target was congruent vs. incongruent in all regions under invalid conditions (all *p* < 0.05) but not under valid conditions (Fig. 4, Table 4). These results indicated that the influence of contextual processing on the neural activity for attentional orienting was observed under invalid conditions but not under valid conditions.

**Table 4:** ROI results.

Regions defined by locating local maxima			
Region	Interaction *F*	Valid incongruent > congruent	Invalid incongruent > congruent
Left TPJ	4.392*	0.004	15.308**
Left IFG	10.082**	3.715	8.956**

ROIs represent previously examined areas that exhibited a significant interaction between context and validity conditions in a 2 × 2 ANOVA (with a voxel-wise spatial extent threshold at *p* < 0.05, FWE corrected, and an intensity threshold at *p* < 0.001, uncorrected). *p < 0.05, **p < 0.01.

## Discussion

We examined attentional orienting by gaze and arrow cues under a localising task in which participants were asked to indicate whether the target (voice and tone) was heard on the left or right side of the headphones. The combination of cues and targets varied across trials, reflecting contextual relationship processing, where the contextual meaning of the cue-target was congruent (social gaze and social voice and arrow and tone) or incongruent (social gaze and tone and arrow and social voice). Although the behavioural results showed no difference between gaze and arrow cues, a RT benefit was observed for targets matching the context of the cue under the invalid condition. This finding suggested that a disengagement of attention from cued locations was facilitated through contextual processing. Previous studies^23, 25^ have demonstrated that attentional orienting can be influenced by contextual processing when targets were presented in a specific context (e.g., colour or emotion) for gaze or arrow cues. Compared with a previous study^23^ in which attentional orienting based on a contextual effect for gaze and arrow was investigated in the context of colour (i.e., schematic white/black eyes as the cue and a black square as the target), the present study examined attentional orienting through contextual processing using facial gaze and voice, which seems to more closely resemble a real-world environment. Furthermore, although another study^25^ examined attentional orienting through gaze cues influenced by emotional context for 80 different images (e.g., a chimney image) as targets, the present study only manipulated two sounds as targets, potentially easing the establishment of a pairing between the cue and target. That is, the pairing of gaze and voice was easily established, and arrow and tone represented the other pair. Thus, the present study observed attentional orienting through contextual processing when using gaze and arrow as cues. Based on these findings, the results of the present study extended those of previous studies^23, 25^, indicating that attentional orienting through centrally presented cues, irrespective of cue characteristics (e.g. social or non-social), could also be modulated by contextual relationship processing between the cue and target.

Importantly, consistent with the behavioural results, the main analyses of fMRI data revealed that neural substrates were not different in response to the contextual relationship processing of cue-target for gaze and arrow cues. Previous studies^28–31^ have revealed that neural substrates in the regions of the TPJ and STS were associated with contextual processing. Consistently, to highlight the neural underpinnings of RT modulated by contextual processing, the results of the present study also demonstrated that the left STS and bilateral TPJ were specifically involved in contextual processing under both gaze and arrow conditions. The results also indicated that these regions were weakly activated when the relationship of cue-target was congruent (the expected target matching the context of the cue) compared with incongruent (the expected target non-matching the context of the cue). We suggest that these regions may be the locations of an inhibitory mechanism, which enhanced neural activity to suppress the processing of incongruent predictions for targets from cue stimuli. Given that the activity of these regions did not differ between social gaze and arrow cues, we further suggest that a comparable neural system was elicited by contextual processing for gaze and arrow cues and further influenced the attentional process. This idea is consistent with previous studies^12–18^ that demonstrated the differences in attention to social and non-social cues were quantitative rather than qualitative.

Interaction analyses in behavioural results indicated that a different pattern of attentional orienting by contextual processing was elicited for valid and invalid conditions. That is, a RT benefit was observed for targets matching the context (e.g. social) of the cue under invalid conditions, but not under valid conditions. We propose that the different patterns between valid and invalid conditions may be influenced through the overlap of the time window between contextual processing and attentional orienting. Electrophysiological studies have demonstrated that the activity of temporal staging differed between valid and invalid conditions^52–54^. These studies reported an amplitude enhanced at P1 (a positive component at occipital electrode sites between 70 and 100 ms post-target onset) in attentional orienting with gaze as the cue under valid versus invalid conditions, whereas a greater amplitude at P3 (a positive component at central/parietal/midline electrode sites between 300 and 500 ms post-target onset) was observed under invalid versus valid conditions. However, in previous studies^55, 56^, the N300 (a negative component at frontal electrode sites at approximately 300 ms) - N400 (a negative component at central/parietal electrode sites at approximately 400 ms) wave reflected an updating of context information. For example, Demiral *et al*.^55^ observed a stronger N300-N400 effect elicited through a semantic context when the contextual scene was presented before the target in a spatial attention task. Given that the pattern of attentional orienting by contextual processing differed between valid and invalid conditions, we suggest that this finding may be influenced through associations with the activities of temporal staging between the influence of attentional orienting (i.e. the mechanism of valid and invalid conditions) and the contextual processing component. Compared with the early stage (70–100 ms) under valid conditions, the time window of processing overlapped with that of contextual information and attentional orienting under invalid conditions at a later stage (300–500 ms), in which these processes could be integrated to suppress a violation of expectancies (the expected target matching the context of the cue) when the contextual relationship of cue-target was incongruent. In addition, we speculated that varying SOA conditions may influence expectations, and the patterns of attentional orienting by contextual processing for valid and invalid conditions could be modulated in the present experiment. That is, if the target was presented at a short SOA prior to the expectation of the subject, then attentional orienting by contextual processing may not be influenced under valid or invalid conditions, whereas if the target was presented at a long SOA after the expectation of the subject, then attentional orienting by contextual processing might be influenced under invalid conditions.

Furthermore, the results of the interaction analyses in fMRI also demonstrated the influence of contextual processing on neural activity for attentional orienting under invalid but not valid conditions. Such neural activities were observed in the left hemisphere TPJ and IFG, an area in the ventral frontoparietal network that may be responsible for invalidity orienting (for reviews, see refs 10 and 11). As mentioned above, analyses of the fMRI data revealed that the left STS and TPJ were involved in the contextual processing of the relationship between the cue and target. The activity in the left TPJ region overlapped in processing the contextual relationship of cue-target and invalidity orienting in attention. Additionally, consistent with the pattern of neural activity for contextual processing in the left STS and TPJ, we observed that when the relationship of cue-target was congruent versus incongruent based on ROI analyses, less activity was observed in all of these brain regions. This result suggests that from the left TPJ to the IFG in the ventral frontoparietal network, neural signals for contextual processing were transferred to invalidity orienting in attention, in which the intrinsic connection pathway is present among these regions^57^. Compared with incongruent conditions, we suggest that the lower activity in the ventral frontoparietal network when the contextual processing of the relationship between cue and target is congruent under invalid conditions may reflect a disengagement of attention from cued locations at a lower cost, which is readily elicited. This idea may explain the behavioural data obtained under invalid conditions, indicating that a lower cost is associated with processing when the contextual processing of the relationship between the cue and target was congruent versus incongruent; thus, participants could disengage attention from the cued location to rapidly capture a target.

### Implications of the present study

Previous behavioural studies^23–26^ had demonstrated that attentional orienting by gaze or arrow cues could be modulated through contextual processing. Consistent with these studies, the behavioural results in the present study also revealed that RT in attentional orienting was modulated through contextual processing. In particular, we observed that attentional orienting was modulated through contextual processing under invalid conditions. Given that the present study identified the influence of the neural substrates by contextual processing under invalid conditions in ventral frontoparietal networks, we suggest that this finding may account for the behavioural data regarding attentional orienting through contextual processing based on the neurocognitive architecture.

In addition, a behavioural study demonstrated impaired attentional orienting when the cue-target relationship is weak (i.e., incongruent context) in individuals with autism spectrum disorder (ASD)^27^, a finding that raises a question regarding whether individuals with ASD exhibit impairment of gaze-triggered attention because activity is impaired in the neural mechanism in the ventral frontoparietal network. Given that impaired gaze-triggered attention may impede and differentially affect the development of the ability to understand the mental state of another individual in social communication^1^, we suggest that an atypical function in the ventral frontoparietal network, particularly in the processing of contextual information, may be associated with the atypical development of social cognition, further suggesting an important direction for future studies combining brain imaging and treatment interventions for social processing deficits in individuals with ASD.

### Limitations

First, we tested attentional orienting by contextual processing using gaze and arrow as cues with two types of targets (social voice and tone) under only visual-auditory cross-modal conditions. Given the complexity of real life, future studies should examine attentional orienting by contextual processing using two types of targets under visual-visual unimodal or visual-tactile cross-modal conditions in which attentional orienting by contextual processing may also play an important role.

Second, the present study involved two types of contextually related cues and targets. In contrast with faces and voices, which are immediately paired in a congruent context, the pairing of the tone and arrow might be influenced through the increasing number of trials throughout the experiment. To evaluate this possibility, all experimental blocks were divided into two parts (first and last half of the block), and a four-factor repeated-measures ANOVA (block × cue × context × validity) was used to analyse the RTs. Given that a significant 4-way interaction was observed (*F* (1, 21) = 5.09, *p* = 0.04, *η*_*p*_^*2*^ = 0.195), two 3-way repeated-measures ANOVA (block × cue × context) was performed under valid and invalid conditions separately. Although no significant interaction was detected for block conditions under invalid conditions, (all *p* > 0.1), we observed that the main effect of context (*F* (1, 21) = 11.74, *p* = 0.003, *η*_*p*_^*2*^ = 0.36) was significant with faster response to congruent than incongruent conditions (349.0 vs. 361.5 ms), thus indicating that contextual processing between the cue and target was immediately established at both gaze and arrow pairings. However, under valid conditions, although we observed no significant main effect of context (*F* (1, 21) = 1.41, *p* = 0.25, *η*_*p*_^*2*^ = 0.063), a significant interaction of block × cue × context was observed (*F* (1, 21) = 12.67, *p* = 0.002, *η*_*p*_^*2*^ = 0.38). The *post hoc* test revealed a significant difference between context conditions (*p* = 0.045) with a faster response to congruent than incongruent conditions (327.4 vs. 346.9 ms) when using arrows as cues in the last half of the block, thereby indicating that attentional orienting by contextual processing could be influenced by increasing the number of trials throughout the experiment under valid conditions when the tone target was matched with arrow cue. In the present study, attentional orienting was modulated through contextual processing under invalid conditions. Thus, we suggest that this effect may be elicited through immediately established pairing between arrow and tone, rather than increasing the number of trials in the experiment. However, future studies should investigate the mechanism of how contextual processing is influenced by increasing the number of trials through the experiment.

Finally, in the present study, we directly contrasted the context conditions by gaze and arrow cues in invalid and valid trials to reveal differences in attentional orienting. Previous studies^15, 20, 21^ manipulated a neutral cue (e.g. direct gaze) as a baseline condition to examine differences in the neural mechanisms between valid and invalid attentional orienting conditions. However, compared with a non-directional arrow as a neutral cue, Engell *et al*.^19^ suggested that a direct gaze, as a neutral cue, was perceived as directional rather than non-directional, which was problematic in terms of comparing social versus non-social cueing in an fMRI study. Future research should investigate the need for a baseline condition in which the neural gaze and arrow cues involve no spatial information and have the same effect on neural activity, such as closed eyes and non-directional arrows.

## Conclusions

In this study, we observed that the response time in attentional orienting by gaze and arrow cues was modulated through contextual processing between the cue and target when contextually congruent and incongruent under invalid conditions in behavioural studies. Additionally, on the neural level, activity in the left TPJ and STS was observed with attentional orienting by gaze and arrow cues in response to contextual processing of the relationship between the cue and target. However, we did not observe any difference in the neural substrates between social gaze and arrows by contextual processing in attentional orienting. This finding adds further evidence in support of the notion that the differences in attention to social and non-social cues are quantitative rather than qualitative. Importantly, both behavioural and fMRI results indicated that the influence of contextual processing on neural activity for attentional orienting occurred under invalid conditions. Such an increase was observed in the ventral frontoparietal network when the cue and target were incongruent rather than congruent. This finding may provide an explanation for the behavioural data regarding attentional orienting by contextual processing based on the neurocognitive architecture.

## Electronic supplementary material

Supplementary_material
