# Supplementary material for: Human cortical activity evoked by contextual processing in attentional orienting
Source: Sci Rep. 2017 Jun 7;7:2962. doi: 10.1038/s41598-017-03104-1 (PMC5462779; doi:10.1038/s41598-017-03104-1)
Supplement: Supplementary file 1 — Supplementary_material [file 41598_2017_3104_MOESM1_ESM.pdf]

Human cortical activity evoked by contextual processing in attentional orienting

Shuo Zhao[1,2,3], Chunlin Li[4], Shota Uono[5], Sayaka Yoshimura[5], and Motomi Toichi[1,3]

[1]Faculty of Human Health Science, Graduate School of Medicine, Kyoto University, Kyoto 606-8507, Japan

[2]International Research Fellow of the Japan Society for the Promotion of Science, Tokyo 102-0083, Japan

[3]Organization for Promoting Neurodevelopmental Disorder Research, Kyoto 606-8392, Japan

[4]School of Biomedical Engineering, Capital Medical University, Beijing 10069, China

[5]Department of Neurodevelopmental Psychiatry, Habilitation and Rehabilitation, Graduate School of Medicine, Kyoto University, Kyoto 606-8507, Japan

Corresponding author: Chunlin Li, School of Biomedical Engineering,Capital Medical University, You An Men, Beijing 10069, China. E-mail: lichunlin1981@163.com, and Motomi Toichi, Faculty of Human Health Science, Graduate School of Medicine, Kyoto University, 53 Shogoin Kawahara-cho, Sakyo-ku, Kyoto 606-8507, Japan. E-mail: toichi.motomi.4v@kyoto-u.ac.jp

Supplementary Information

Human cortical activity evoked by contextual processing in attentional orienting

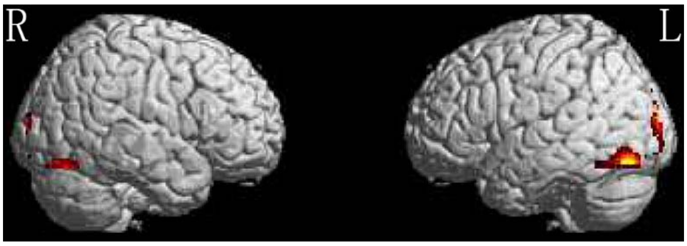

Supplementary Figure 1. Exploratory whole-brain analysis indicating the brain regions significantly activated in response to gaze compared with arrow conditions. Areas of activation are rendered on spatially normalised brains. L, left hemisphere; R, right hemisphere.

Supplementary Table 1

Main effect of gaze cues: GAZE > ARROW

Side	Area	Region	BA	Coordinates			Z-value	P (FWE)	P (FWE)	P (uncorr)	Cluster size
				x	y	z		(cluster level)	(peak level)	(peak level)	(mm^3)
L	Occipital	Fusiform gyrus	19	-36	-76	-16	7.70	0.000	0.000	0.000	3149
	Anterior	Culmen	-	-6	-62	2	7.53		0.000	0.000	
	Occipital	Lingual gyrus	-	-14	-56	-2	5.77		0.000	0.000	
R	Anterior	Culmen	-	8	-70	-8	5.75		0.000	0.000	
	Occipital	Fusiform gyrus	19	36	-68	-18	4.73		0.000	0.000	

BA = Brodmann's area; FWE = family-wise error; A voxel-wise spatial extent threshold at $p < 0.05$, FWE corrected, and an intensity threshold at $p < 0.001$, uncorrected.

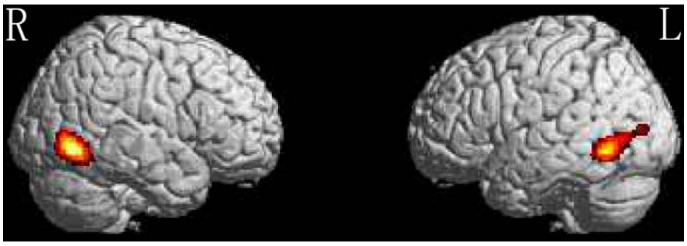

Supplementary Figure 2. Exploratory whole-brain analysis indicating the brain regions significantly activated in response to arrow compared with gaze conditions. Areas of activation are rendered on spatially normalised brains. L, left hemisphere; R, right hemisphere.

Supplementary Table 2

Main effect of arrow cues: Arrow > GAZE

Side	Area	Region	BA	Coordinates			Z-value	P (FWE)	P (FWE)	P (uncorr)	Cluster size
				x	y	z		(cluster level)	(peak level)	(peak level)	(mm^3)
R	Temporal	Middle temporal gyrus	37	44	-62	-8	7.56	0.002	0.000	0.000	684
L	Occipital	Middle occipital gyrus	19	-50	-58	-8	6.46	0.007	0.000	0.000	504

BA = Brodmann's area; FWE = family-wise error; A voxel-wise spatial extent threshold at $p < 0.05$, FWE corrected, and an intensity threshold at $p < 0.001$, uncorrected.

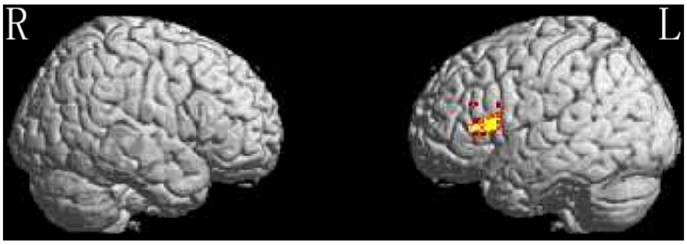

Supplementary Figure 3. Exploratory whole-brain analysis indicating the brain regions significantly activated in response to invalid versus valid conditions. Areas of activation are rendered on spatially normalised brains. L, left hemisphere; R, right hemisphere.

Supplementary Table 3

Main effect of invalid condition: Invalid > Valid

Side	Area	Region	BA	Coordinates			Z-value	P (FWE)	P (FWE)	P (uncorr)	Cluster size
				x	y	z		(cluster level)	(peak level)	(peak level)	(mm^3)
L	Frontal	Inferior frontal gyrus	10/13	-38	26	10	5.744	0.002	0.000	0.000	716
	Limbic	Anterior cingulate	32	-14	24	24	4.162		0.149	0.000	
	Frontal	Middle frontal gyrus	46	-40	14	24	3.195		0.963	0.000	

BA = Brodmann's area; FWE = family-wise error; A voxel-wise spatial extent threshold at $p < 0.05$, FWE corrected, and an intensity threshold at $p < 0.001$, uncorrected.
